# Supplementary material for: An Hfq-dependent post-transcriptional mechanism fine tunes RecB expression in Escherichia coli
Source: eLife. 2025 Aug 12;13:RP94918. doi: 10.7554/eLife.94918 (PMC12342828; doi:10.7554/eLife.94918)
Supplement: Supplementary file 1. [file elife-94918-supp1.pdf]

Supplementary file 1

An Hfq-dependent post-transcriptional mechanism fine tunes RecB expression in *Escherichia coli*

Irina Kalita, Ira Alexandra Iosub, Lorna McLaren, Louise Goossens, Sander Granneman, Meriem El Karoui

---

This Supplementary file includes Supplementary Tables and Supplementary References.

Supplementary Tables **2**

Table A: *E. coli* strains used in the study . . . 2
Table B: Plasmids used in the study . . . 3
Table C: Primers used for strain and plasmid construction . . . 4
Table D: The sequence of the gBlock used for the construction of the *recB*-5′UTR strain . . 5
Table E: Oligos used for RT-qPCR quantification . . . 6
Table F: Sequences of *recB* RNA FISH probes . . . 7
Table G: Parameters of the model of RecB expression . . . 8

Supplementary References **9**

Supplementary Tables

Table A: *E. coli* strains used in the study.

**Strain**	**Genotype**	**Source**
MG1655	$F^- \lambda^-$ *ilvG*$^-$ *rfb-50 rph-1*	Lab stock
BW27783	$F^- \lambda^-$ Δ*(araD-araB)567* Δ*lacZ4787*::rrnB-3 Δ*(araH-araF)570(::FRT)* Δ*araEp-532::FRT* ϕP_{cp8}*araE535 rph-1* Δ*(rhaD-rhaB)568 hsdR514*	Lab stock
MEK65	MG1655 *recB165*::*halotag*	[1]
MEK1326	MG1655 Δ*recB*	[1]
MEK1329	BW27783 Δ*recB*	This work
MEK1902	MG1655 Δ*hfq*	This work
MEK1457	MG1655 *recB165*::*halotag* Δ*hfq*	This work
MEK1888	MG1655 Δ*chiX*	This work
MEK1449	MG1655 *recB165*::*halotag* Δ*chiX*	This work
MEK1938	MG1655 *recB165*::*halotag* 5′UTR-*recB* $\Delta(TTAA...TGAT)_{36nt}$	This work

Table B: Plasmids used in the study.

Plasmids	Description	Source
pTOF24Δ*recB*	pTOF24-derivative plasmid used for construction of Δ*recB* strain	[2] [1]
pTOF24 *recB*-5′UTR	pTOF24-derivative plasmid used for construction of *recB*-5′UTR strain	This work
pBAD33	Backbone plasmid used for construction of pIK02	[3]
pIK02	pBAD33-derivative plasmid carrying *recB* gene under control of arabinose-inducible promoter, P*araBAD*	This work
pZA21MCS	Backbone plasmid used for construction of pZA21-ChiX	Expressys
pZA21-ChiX	pZA21MCS-derivative plasmid carrying *chiX*	This work
pZA21-CyaR	pZA21MCS-derivative plasmid carrying *cyaR*	[4]
pDWS2	pBR322-derivative plasmid carrying native *recC-ptrA-recB-recD* chromosomal region	[5] [2]
pQE80L	Backbone plasmid used for construction of pQE-Hfq	[6] [3]
pQE-Hfq	pQE80L-derivative plasmid carrying *hfq*	[6] [3]
pQE-Hfq(Y25D)	pQE80L-derivative plasmid carrying *hfq(Y25D)*	[6] [3]
pQE-Hfq(K56A)	pQE80L-derivative plasmid carrying *hfq(K56A)*	[6] [3]

[1] Gift from Prof. David Leach, The University of Edinburgh
[2] Gift from Prof. Gerald Smith, The Fred Hutchinson Cancer Research Center
[3] Gift from Prof. Teppei Morita and Prof. Hiroji Aiba, Suzuka University of Medical Sciences

Table C: Primers used for strain and plasmid construction.

Primer ID	Sequence (5′-3′)
hfq_H1_P1	GAATCGAAAGGTTCAAAGTACAAATAAGCATATAAGGAAAAGAG AGAATGGTGTAGGCTGGAGCTGCTTC
hfq_H2_P2	CTCCCCGTGTAAAAAAACAGCCCGAAACCTTATTCGGTTTCTTCGC TGTCGGTCCATATGAATATCCTCCTTAG
ChiX_H1_P1	TCTTGCCTAAGAGTATTGGCAGGATGGTGAGATTGAGCGACAATC GAGTTGTGTAGGCTGGAGCTGCTTC
ChiX_H2_P2	CACCTGTATGGAGAAGGGAATTTGCCGCAAATGTTGCGCTAAAAA AATGGCGGTCCATATGAATATCCTCCTTAG
chiX_ZA21	ACACCGTCGCTTAAAGTGACGGCATAATAATAAAAAAATGAAATT CCTCTTTGACGGGCCAATAGCGATATTGGCCATTTTTTTGGTACGCG TGCTAGAGGCATC
pZA21_5P	5P-GTGCTCAGTATCTCTATCACTGA
oIK01	AAGCTTGGCTGTTTTGGCGGATGAG
oIK02	ACCGAGCTCGAATTCGCTAGCCCAAAA
oIK03	ACCCGTTTTTTTGGGCTAGCGAATTCGAGCTCGGTCCTGATGAGTG AAAAGAATGAGTG
oIK04	GAAAATCTTCTCTCATCCGCCAAAACAGCCAAGCTTCTCCACAGCT TCCAGTAATTGC

Table D: The sequence of the gBlock used for the construction of the *recB*-5′UTR strain.

5′-GTAATACAAGGGGTGTTATGAGCCATATTCAACGGGAAACGTCTTGCTCGAGGGTTCAG<br>CAATAGCATCGGCCAGGCGGTCTACCGCACCAGGCAAGGCGTCGTTCTCAACTTCCAGAT<br>AGAAAGCCGTGCGATACGGCGCAGTGCTGGCATTGTGACTACCGCCGTGCATTTTGAGAT<br>ATTCGGCCAGACTGTCAGCCTGCGGGTACTTTTTCGACCCCATCAGACTCATATGTTCAAG<br>GTAATGTGCCAGCCCCTGGTACGCCTCGGGATCTTCCAGCGACCCAACGGGCACCACCAG<br>CGCCGAGAGCGATTTAACTGCCTGCGGATCAGAAACCAGCAAGACCACCATACCGTTATC<br>CAGACGTATAGCCTGATACTGGCGGTTATCTTTATCACTTTTACGGATGGTTTCCTGAATCG<br>GCTGCCATCCCGTTTCTGCCTGACTTAAGGGTGCCCAAAGGGCAACTAACAACAATAATG<br>CTTTGAACCAGGTGCTGCGGGGCATTCACGGACCTCATAAGCTTCGCAAATCATCTGCCA<br>GAATTTAATCTTGTGCTGCACGAGTCAGCCTATGTTTATATAACCATCAGTCCGTGACTGGT<br>GCGCATCATAAAGTAAGCGGATAGATTGCGCAATTTTTATACAGCACTCATGACTGATTAA<br>AGCGAAACAGCGGTAACAGGAAACGTTGCGACTGTTCAACGATGGCCTCCATTGTCTCTG<br>GTGTTAATTGCCGCCAGAGCCTTTGATACCAGATATCATCACCTTCGCCACGCACCATCAT<br>GTTGCCTTCGTAAGCCTGAAGGAATTTCGTACGGGCTTTTTGCAACGTGGAATCGTCATCC<br>AGCATGGCATCGTTTTGCGCGTCATAACAGGTTTTTAGCCACGCGCCGCCACTTTCAGGTA<br>ACACCAGCAATGGCGCGGACATTCCTTCACGATACCCCTCAATCAGTTGTGAGAGGTAAT<br>GCAAAGCCTGTTCGGCGTCGACCGGTGGCGAATGGGACGCGCCCTGTAGCGGCGCATTA<br>AGCGCGGC-3′

Table E: Oligos used for RT-qPCR quantification.

**Sequence (5′-3′)**	**Gene**
TGGCCTGACGCGTATGTTGT	*recC*
TGCCCCACCAGTTCTGCAAT	*recC*
CTCTTGCGGGTTACGGACGT	*recD*
ATTCAGTCCAGCCACGCCAA	*recD*
TCTGGCTTCATCGCTCGCAA	*ptrA*
TGGGGCATGGGCTTCCTTTT	*ptrA*
ACCCGCGCATTGGCTGAGAT	*recB*
CACCGCTTTCGCTACGCAGC	*recB*
CGGTGGTCCCACCTGACC	*rrfD*
CCTACTCTCGCATGGGGAGACC	*rrfD*
CGTCTCGCCCGGTTTCTCAT	*hfq*
GGAAGTATTCTGCGCGCTGC	*hfq*
GTCGCTTAAAGTGACGGCAT	*chiX*
TCGCTATTGGCCCGTCAAAGA	*chiX*
GCTAGCTGTACCAGGAACCACC	*cyaR*
GGGAGATTACACAGGCTAAGGAGG	*cyaR*

Table F: Sequences of *recB* RNA FISH probes labelled with TAMRA dye.

Probe ID	Sequence (5′-3′)
RecB-TAMRA_1	ctgtaagggcaagcgcaaag
RecB-TAMRA_2	gcaatcgtaaaggttttgcc
RecB-TAMRA_3	tcgtggatattgctacggat
RecB-TAMRA_4	gttcggctaacaacaaccac
RecB-TAMRA_5	aaaggcattcaggttgagca
RecB-TAMRA_6	aatcagctgctgctcaaaca
RecB-TAMRA_7	aaagacgacctgggctattt
RecB-TAMRA_8	cgccttgcagataacgatta
RecB-TAMRA_9	ctgctgttttaccgtatcaa
RecB-TAMRA_10	accagaagattcgatcagcg
RecB-TAMRA_11	cggttaaacttgcgtcgatc
RecB-TAMRA_12	atcttgtcgatccatttagc
RecB-TAMRA_13	ccggcaactgataactgttt
RecB-TAMRA_14	cttcgtgcgatcttctaaga
RecB-TAMRA_15	ttgatcgatcgcctcaaaca
RecB-TAMRA_16	cagatcgcggatcgacaatg
RecB-TAMRA_17	agccgacttaacatgtcatc
RecB-TAMRA_18	cgccaattagcaacaatgcg
RecB-TAMRA_19	tacgcgccttcatataagtg
RecB-TAMRA_20	tgtctaaagtgtagtgggcg
RecB-TAMRA_21	aagcttattcacgctgttca
RecB-TAMRA_22	tcgcgaaacatgaacgcgtc
RecB-TAMRA_23	aaacggaagggatttccagc
RecB-TAMRA_24	cctgcaacaaccaaagcatt
RecB-TAMRA_25	cagatttgccgataaccatc
RecB-TAMRA_26	gttttcagcaatgttacgcg
RecB-TAMRA_27	ttgtagcagttcgctgatat
RecB-TAMRA_28	tggatcgtgacaatctgcac
RecB-TAMRA_29	tggacgcggaaattggtgat
RecB-TAMRA_30	atcgtgataaaacgcctgct
RecB-TAMRA_31	ttaagatccagaactgcctc
RecB-TAMRA_32	aagcaaacgcagatcttccg
RecB-TAMRA_33	aatgccaaaccgaacgtgtc
RecB-TAMRA_34	aacgcttcaatacaggtgcg
RecB-TAMRA_35	ttggttatcaccagtttgtg
RecB-TAMRA_36	tcagctctgctgtagaaaca
RecB-TAMRA_37	aaaccagagtagctggtgac
RecB-TAMRA_38	tgatgtggtgttaacgtcgg
RecB-TAMRA_39	tcaaccggctgggtaaaatc
RecB-TAMRA_40	ttattgcgggcggaaagttg
RecB-TAMRA_41	gataaaactccatctccacc
RecB-TAMRA_42	aacgtatcaagctgactggc
RecB-TAMRA_43	gccagggtataaagctgata
RecB-TAMRA_44	caatgcgatggcgcagataa
RecB-TAMRA_45	tggtgctcatagtcgtaatc
RecB-TAMRA_46	acagataaataacgccgcca
RecB-TAMRA_47	gatgttctttatcaacgcca
RecB-TAMRA_48	cataccggcaaacatctcat

Table G: Parameters of the model of RecB expression.

Parameter	Meaning	Value	95% CI	Source
k_m	transcription rate	0.21 min^{-1}	[0.13 0.33] min^{-1}	Estimated
γ_m	mRNA degradation rate	0.62 min^{-1}	[0.48 0.75] min^{-1}	Measured
b	mRNA burst size	0.95 molec	[0.76 1.19] molec	Estimated
k_p	translation rate	0.15 min^{-1}	[0.12 0.18] min^{-1}	Estimated
γ_p	protein removal rate	0.015 min^{-1}	[0.011 0.019] min^{-1}	Measured

Supplementary References

[1] A Lepore et al. "Quantification of very low-abundant proteins in bacteria using the HaloTag and epi-fluorescence microscopy". In: *Sci Rep* 9.7902 (2019). DOI: 10.1038/s41598-019-44278-0.

[2] E Darmon et al. "*E. coli* SbcCD and RecA Control Chromosomal Rearrangement Induced by an Interrupted Palindrome". In: *Mol Cell* 39.1 (2010), pp. 59–70. DOI: 10.1016/j.molcel.2010.06.011.

[3] LM Guzman et al. "Tight regulation, modulation, and high-level expression by vectors containing the arabinose PBAD promoter". In: *J Bacteriol* 177.14 (1995), pp. 4121–30. DOI: 10.1128/jb.177.14.4121-4130.1995.

[4] IA Iosub et al. "Hfq CLASH uncovers sRNA-target interaction networks enhancing adaptation to nutrient availability". In: *eLife* 9 (2020), e54655. DOI: 10.7554/eLife.54655.

[5] AS Ponticelli et al. "Chi-dependent DNA strand cleavage by RecBC enzyme". In: *Cell* 41.1 (1985), pp. 145–51. DOI: 10.1016/0092-8674(85)90069-8.

[6] T Morita and H Aiba. "Mechanism and physiological significance of autoregulation of the *Escherichia coli hfq* gene". In: *RNA* 25.2 (2019), pp. 264–76. DOI: 10.1261/rna.068106.118.
